# Recent Advances in the Development of Immunoproteasome Inhibitors as Anti-Cancer Agents: The Past 5 Years

**DOI:** 10.3390/molecules30030755

**Published:** 2025-02-06

**Authors:** Francesca Mancuso, Carla Di Chio, Francesca Di Matteo, Gerardina Smaldone, Nunzio Iraci, Salvatore Vincenzo Giofrè

**Affiliations:** 1 Department of Chemical, Biological, Pharmaceutical and Environmental Sciences (CHIBIOFARAM), University of Messina, Viale F. Stagno d’Alcontres 31, 98166 Messina, Italy; framancuso@unime.it (F.M.);; 2 Department of Pharmacy, University of Salerno, Via G. Paolo II, 84084 Fisciano, Italy

**Keywords:** immunoproteasome, cancer, covalent inhibitors, non-covalent inhibitors, peptide backbone, non-peptide inhibitors, structure–activity relationship

## Abstract

The immunoproteasome (iCP) is an isoform of the 20S proteasome that is expressed in response to cellular stress or inflammatory stimuli. The primary role of the iCP is to hydrolyze proteins into peptides that can be loaded into the MHC-I complex. Beyond its primary role in the adaptive immune response, it is also involved in the pathogenic mechanism of numerous disease states such as inflammatory conditions and cancer. In the last decade, a huge number of immunoproteasome-specific inhibitors have been described, allowing researchers to elucidate the role of the immunoproteasome as a potential therapeutic target for these diseases. The present manuscript summarizes the latest advances regarding immunoproteasome inhibitors tested against different cancer models. Specifically, it will focus on peptide and non-peptide analogs that have been reported in the last five years, together with their structure–activity relationship (SAR) studies. It aims to provide structural insights into this class of compounds pertaining to their favorable applicability as selective iCP inhibitors in the treatment of cancer.

## 1. Introduction

The ubiquitin–proteasome system (UPS) is a non-lysosomal system involved in the degradation of damaged or misfolded ubiquitin-tagged proteins [1]. The 26S proteasome is formed of two regulatory caps (19S) and a central core (20S). The first two are responsible for the recognition of ubiquitin-tagged proteins and for the transport of these proteins toward the core, which is responsible for the proteolytic activity. It has a typical barrel-like structure and is formed by four superposed heptameric rings. The two outer rings (α rings) are composed of α subunits only and have a structural function, while the inner rings (β rings) consist of β subunits and exert a proteolytic function [2].The β rings contain three catalytic subunits with different proteolytic activities: the β1 subunit (caspase-like, C-L) cleaves the peptide bond after basic residues, the β2 subunit (trypsin-like, T-L) cleaves after acid residues and the β5 subunit (chymotrypsin-like, ChT-L) cleaves after hydrophobic residues [3,4]. In vertebrates, there are several isoforms of the proteasome: the constitutive proteasome (cCP), located in all cells, the immunoproteasome (iCP), mainly expressed in immune cells, and the thymoproteasome (tCP), only expressed in cortical epithelial cells of the thymus [5]. The immunoproteasome is expressed following interferon-γ (INF-γ) stimulation, tumor necrosis factor-α (TNF-α) stimulation or viral infection. The iCP replaces the catalytic subunits of the constitutive proteasome with β1i, β2i and β5i, in which the subunits β2i and β5i maintain the same proteolytic activity, while subunit β1i exhibits chymotrypsin-like activity.

**Figure 1:**
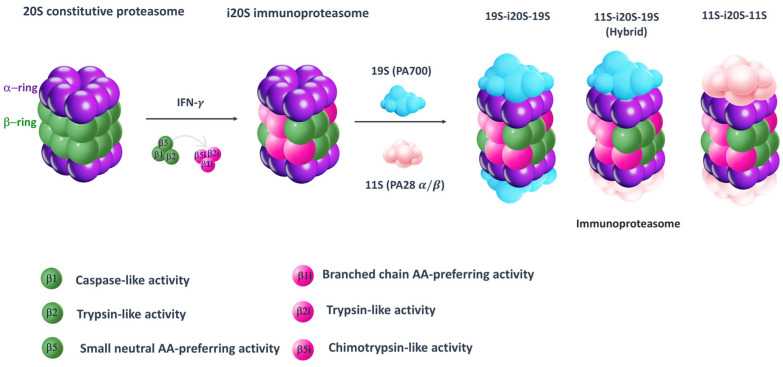
Simplified model for 20S constitutive proteasome and immunoproteasome. The 20S proteasome is a cylindrical structure consisting of two outer α (purple) and two inner β rings (green). As result of interferon-γ (IFN-γ) stimulation, proteasome subunits β1, β2 and β5 are replaced by their corresponding immune subunits: β1i, β2i and β5i. Then, the mature i20S binds to either 19S (PA700) or 11S (PA28 α/β), or a combination of them, to assemble into three different types of immunoproteasomes (19S-i20S-19S, 11S-i20S-19S and 11S-i20S-11S).

In the proteasome structure, even regulatory particles are key components, whose binding to the outer α subunits induces the opening of the α gate, allowing for the entrance of the substrate proteins into the catalytic core. The second most common regulatory particle, after 19S (PA700), is 11S (PA28 α/β), which preferentially binds immunoproteasomes. Indeed, when the mature iCP is formed, it binds to the regulatory particles 19S (PA700) or 11S (PA28 α/β), or a combination of them, to assemble into three different types of immunoproteasomes (19S-i20S-19S, 11S-i20S-19S and 11S-i20S-11S) (Figure 1).

The primary role of the immunoproteasome is to process the antigen peptide so that it can be introduced into type I major histocompatibility complexes (MHC-Is), although it is also involved in the degradation of damaged proteins as the constitutive isoform [6]. Several studies have shown that the immunoproteasome is involved in cell differentiation and proliferation in different tissues [7,8,9]. Indeed, one study reported that iCP was shown to regulate the immune response and cell division by modulating gene transcription factors such as IRF3, IRF7, STAT1, STAT3 and STAT6 [10].

Considering its activity, it has been shown that modulation of the immunoproteasome may be a good strategy for the treatment of various diseases, such as cancer [11], autoimmune disorders [6] and neurodegenerative diseases [12].

Unlike several articles that have thoroughly reviewed immunoproteasome inhibitors and their potential use in different pathological conditions over the years [13,14,15], this review will narrow its focus to immunoproteasome inhibitors tested against different cancer models, as the immunoproteasome is overexpressed in several tumor forms [11]. Moreover, particular emphasis has been given to the inhibitors’ structure–activity relationships in an attempt to give an updated overview of the molecular determinants that might influence potency and selectivity.

The environment of tumor cells is very different from that of healthy cells. In fact, there is an influx of lymphocytes that release high amounts of INF-γ, stimulating the production of iCP [9,10,11,16]. This type of phenomenon is particularly common in solid tumors, such as breast and colorectal cancer [17,18]. On the contrary, in hematological tumors, such as multiple myeloma (MM) and leukemia, iCP overexpression is not related to stimulation by lymphocytes, as hematopoietic cells directly express the immunoproteasome [19,20]. The use of immunoproteasome inhibitors could be a good therapeutic strategy. In particular, the selective inhibition of the β5i subunit or the simultaneous inhibition of the β1i and β5i subunits could find applications in antitumor treatments [15,21].

## 2. Immunoproteasome Inhibitors in Cancer Therapy

**Figure 2:**
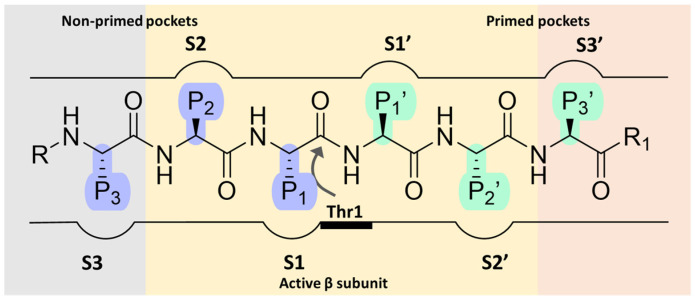
Schematic representation of proteasome substrate-binding channel with the non-primed (S) and the primed (S’) pockets occupied by the side chains (P sites) of a generic peptide.

Over the past two decades, extensive efforts have been made to search for proteasome inhibitors that could serve both as molecular probes to investigate proteasome biology and as potential therapeutic agents for treating various pathological conditions, including cancer. The fulcrum of proteasome inhibition is represented by the substrate-binding channel with its relative pockets (S pockets) as well as the *N*-terminal threonine residues (Thr1s) in the active site. Specifically, as a member of the endo-protease family, the substrate-binding channel consists of primed and non-primed specificity pockets (S pockets) that bind polypeptides in the *C*-to-*N*-terminal direction. Most of the known proteasome inhibitors are peptide-like derivatives with side chains (P sites) opportunely selected to gain selectivity by fitting into the corresponding S pockets (Figure 2).

The non-primed sites allow close interaction with the *N*-terminal polypeptide portion, thereby determining cleavage specificity. In contrast, the primed pockets play an ancillary role by participating in the release of the *C*-terminal cleavage products during the first part of the reaction cycle [22]. The scissile peptide bond is cleaved by the Thr1 residue (Figure 2) located between the primed and the non-primed pockets, thus classifying the proteasome as an *N*-terminal nucleophile hydrolase enzyme.

Most inhibitors target the β5 subunit, whose inactivation has the greatest impact on general proteolysis, whereas inhibition of β1 and β2 subunits has less influence on protein breakdown rates [23]. The majority of β5 inhibitors feature an electrophilic warhead that binds, either reversibly or irreversibly, to the active site Thr1.

### 2.1. Non-Selective Immunoproteasome Inhibitors in Clinical Uses and Trials

**Figure 3:**
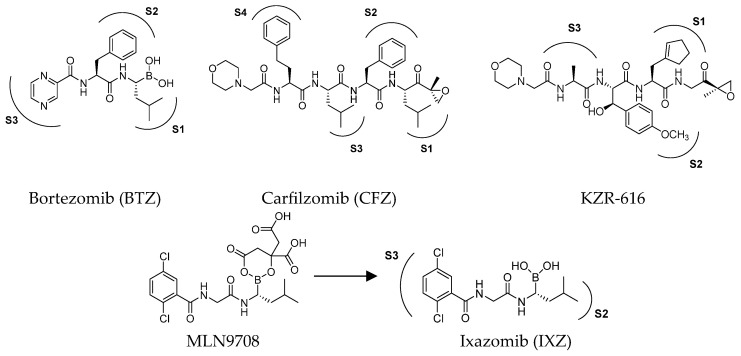
Structures of FDA-approved proteasome inhibitors.

Efforts over time have led to the identification of several small-molecule proteasome inhibitors, including the FDA-approved bortezomib, carfilzomib and ixazomib (Figure 3). Most of the known proteasome inhibitors are peptide-based analogs bearing an electrophilic warhead, such as a boronic acid, an epoxyketone or a β-lactone, at the *C*-terminus. These mechanism-based inhibitors are attacked by the nucleophilic hydroxyl side chain of the Thr1 residue of the catalytic β subunits. All clinically approved proteasome inhibitors, or those under clinical evaluation, exhibit inhibitory activity against multiple active sites of both cCP and iCP, making them broad-spectrum proteasome inhibitors. An exception to this is KZR-616, which is the only selective immunoproteasome inhibitor advanced to clinical trials, for the treatment of systemic lupus erythematosus [24]. Due to the ubiquitous expression of cCP, non-selective inhibition of both cCP and iCP is believed to be responsible for the occurrence of side effects, which have undoubtedly limited the therapeutic application of these drugs.

**Figure 4:**
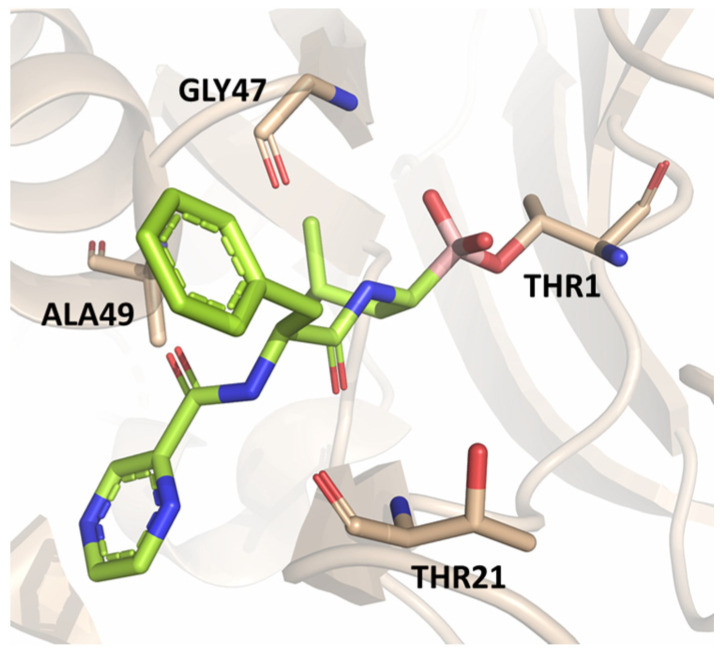
Crystal structure of the yeast 20S proteasome in complex with bortezomib (PBD ID: 2F16).

The first approved proteasome inhibitor, bortezomib (BTZ, previously known as PS-341), was approved by the US Food and Drug Administration (FDA) in 2003 for the treatment of multiple myeloma and mantle cell lymphoma. It is a reversible dipeptide boronic acid analog that inhibits the catalytic activities of both the constitutive proteasome and the immunoproteasome by forming a slowly reversible covalent boron–oxygen bond with the tetrahedral adduct of the γ-OH of the Thr1 residue in the *N*-terminal β5 subunit of the proteasome (Figure 4). Specifically, bortezomib targets the chymotrypsin-like subunits of β5c and β5i with low nanomolar IC_50_ values of 7 nM and 4 nM, respectively. It is less potent against other subunits with IC_50_ values ranging from 1.5 to 4.2 μM. Although bortezomib has demonstrated great efficacy in the treatment of hematological disease, the results have been less satisfactory for solid tumors, and its administration is limited by a narrow therapeutic window. Furthermore, bortezomib must be administered intravenously and is associated with notable side effects like thrombocytopenia, peripheral neuropathy and gastrointestinal disorders [25].

The clinical success and the shortcomings of bortezomib have prompted the search for new proteasome inhibitors with lower toxicity and a greater therapeutic index. This led to the development of carfilzomib (formerly PR-171, Figure 3), a second-generation proteasome inhibitor approved by the FDA in 2012 for the treatment of multiple myeloma. Chemically, it is a tetrapeptidyl α’,β’-epoxyketone-based analog, originally derived from the natural compound epoxomicin, which covalently attacks active site Thr1, resulting in the formation of a morpholine ring [26]. It targets the β5c and β5i subunits with IC_50_ values of 6 nM and 33 nM, respectively, while the IC_50_ values against other subunits are higher than 600 nM, demonstrating an improved selectivity profile over bortezomib, with fewer off-target effects [27].

The first orally available proteasome inhibitor approved by the FDA was ixazomib (Figure 3), which has been used in combination with dexamethasone and lenalidomide for the treatment of MM since 2015. It is administered as the prodrug MLN9708, which undergoes rapid hydrolysis to form the bioactive boronate (Figure 3), targeting the β5c subunit with an IC_50_ value of 3.4 nM [28]. However, no data are available to date concerning its inhibitory activity on the immunoproteasome.

**Figure 5:**
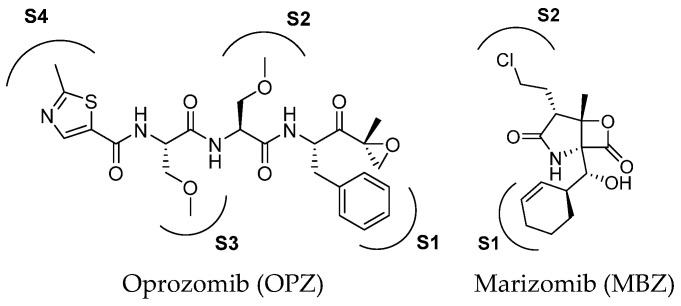
Two-dimensional structure of proteasome inhibitors in clinical trials.

Oprozomib and marizomib (Figure 5) are non-selective proteasome inhibitors currently under phase Ib/II and III clinical evaluation, respectively. Oprozomib (ONX-0912) was identified in a systematic SAR study aimed at discovering orally bioavailable carfilzomib analogs.

**Figure 6:**
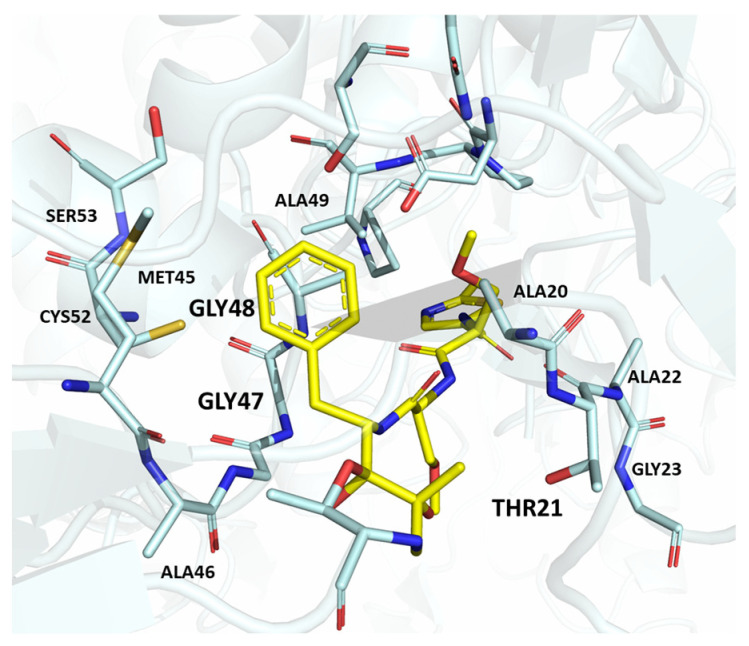
Human 26S proteasome in complex with oprozomib (PBD ID: 5M32).

The high-resolution X-ray co-crystal structure revealed that the epoxyketone moiety of oprozomib reacts with the Thr1 Oγ and Thr1 *N*, resulting in the formation of a seven-membered ring adduct 1,4-oxazepane (Figure 6) [29]. The possibility of delivering oprozomib via oral administration, combined with its efficacy against MM cells resistant to bortezomib or to other conventional therapies, has generated great interest in this novel inhibitor [30]. Although oprozomib is structurally unrelated to bortezomib, it possesses the same ability to predominantly inhibit the ChT-L β subunits β5c and β5i, with IC_50_ values of 36 and 82 nM, respectively.

The discovery of the novel anti-cancer agent marizomib (previously known as NPI-0052) resulted from the exploration of marine environments for the discovery of new natural compounds for drug development [31,32]. Marizomib has been reported as a highly potent inhibitor of cCP and tCP and it is also known to inhibit the iCP, although its potency against specific subunits is not well defined. The high potency of marizomib has been attributed to its unique substituent on the bicyclic system, specifically to the presence of the C2 chloroethyl moiety. Like other PIs, MZB demonstrates significant antitumor activity in preclinical models of various malignancies. Furthermore, it is characterized by high blood–brain barrier permeability, making it a promising tool for glioblastoma management [33].

### 2.2. Selective Immunoproteasome Inhibitors

Similarly to cCP inhibitors, most of the reported iCP inhibitors harbor short peptide structures. Non-peptidic iCP inhibitors have also been reported and are endowed with a wider chemical diversity and optimization potential. This is crucial for expanding the structural classes of known iCP inhibitors and identifying new lead compounds with improved pharmacokinetic properties for future clinical application. Due to the large number and chemical diversity of iCP inhibitors reported in recent years, they will be grouped below based on their mechanism of action and chemical classes. Furthermore, only derivatives with described and verified antitumor activity will be discussed.

Advances in knowledge regarding immunoproteasome structure have shed light on the main structural differences between β5c and β5i, making the rational design of potential selective immunoproteasome inhibitors more feasible. Looking at the P1 site of both β5c and β5i, the residues responsible for ChT-L activity, i.e., Ala20, Met45, Ala49 and Cys52, are conserved. 

In addition to this variation in AA sequence, the S1 pockets of β5c and β5i differ in their size. The conformation assumed by residue Met45 in β5i widens the S1 pocket as a result of strong van der Waals interactions between Met45 and the side chain of Gln53. Oppositely, the stabilization of Met45 in β5c subunits is hampered by the replacement of Gln53 (β5i) with serine. The residues Lys32 in β5c and Asn32 in β5i also contribute to the differing architecture of the S1 sites. As a result, the S1 pocket of β5c ideally accommodates peptides with small amino acids (Ala or Val), while the S1 pocket of β5i favors large hydrophobic residues (Tyr, Trp or Phe).

Therefore, selective β5i inhibitors require bulky hydrophobic groups in P1. Due to substitution of Ala27 in β5c with Ser27 in β5i, the introduction of small, hydrophilic P3 groups in novel potential β5i inhibitors could be beneficial [15,34]. Furthermore, the S2 pocket of β5i is smaller compared to β5c due to the substitution of the constitutive subunit Gly48 residue with Cys or Ser in β5i, depending on the species.

#### 2.2.1. Covalent Peptidyl Immunoproteasome Inhibitors

Most of the inhibitors reported so far are peptide epoxyketones or sulfonyl fluorides that act covalently and irreversibly within the active site. However, irreversible and covalent binding can induce liver toxicity, even when off-target binding occurs at low levels. Additionally, irreversible inhibitors often lead to reduced selectivity in the long run.

**Figure 7:**
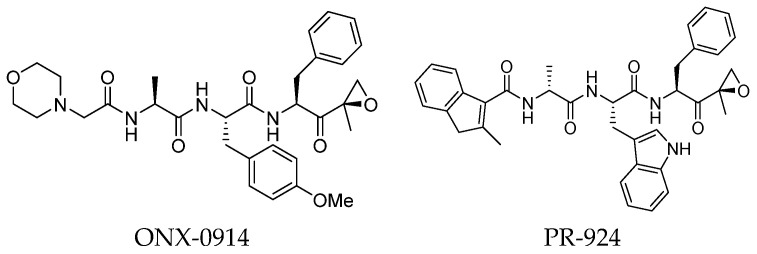
Structure of the epoxyketone peptides ONX-0914 and PR-924.

Undoubtedly, the most popular class of iCP inhibitor is the covalent epoxyketone peptides, among which ONX-0914 (formerly PR-975, Figure 7), with moderate β5i selectivity (β5i, IC50 = 5.7 nM; β5c, IC50 = 54 nM; β5c/β5i ratio: 9.0), was the first to be identified in 2009. A plethora of preclinical studies demonstrated the potential of ONX-0914 as a treatment for autoimmune diseases and chronic inflammation. Furthermore, recent scientific evidence has highlighted the ability of ONX-0914 to significantly reduce the growth of acute lymphoblastic leukemia tumors and multiple myeloma cells both in vitro and in vivo [35,36]. The biological effects of ONX-0914 were initially attributed to the selective inhibition of the β5i subunit. However, more recent studies have shown that co-inhibition, or at least a partial blockage, of a second site (β1i) is necessary for its anti-inflammatory properties. On this basis, although ONX-0914 was initially classified as a β5i-selective inhibitor, it has been recently reclassified as a pan-iCP inhibitor [14].

In 2009, Proteolix Inc. also reported a β5i-selective tripeptide epoxyketone inhibitor named PR-294 (Figure 7), which demonstrated high potency and selectivity for the β5i subunit (β5i, IC_50_ = 0.022 µM; β5c, IC_50_ = 2.9 µM; β5c/β5i ratio: 131). PR-924 was shown to inhibit growth and induce cell death in multiple myeloma and primary patient-derived MM cells, without significantly affecting normal peripheral blood mononuclear cells [37].

ONX 0914 and PR-924 share a common bulky phenylalanine residue at the P1 site, which drives the selectivity of these peptides for β5i through its interaction with the S1 pocket. Due to their interesting selectivity profiles, both compounds have been further investigated over the past five years by various academic groups in the search for novel potential iCP inhibitors (Section 3).

KZR-616 (Figure 3) is an epoxyketone peptide developed to improve the pharmacokinetic profile of ONX 0914, which was precluded from clinical trials due to its low solubility [38]. KZR-616 is the first selective immunoproteasome inhibitor to reach clinical trials for the treatment of autoimmune diseases [39,40].

#### 2.2.2. Non-Covalent Peptidyl and Non-Peptidyl Immunoproteasome Inhibitors

In recent years, several non-covalent iCP inhibitors, both peptidyl and non-peptidyl in structure, have been reported. Among these, a variety of non-covalent *N*,*C*-capped dipeptide and dipeptidomimetic inhibitors with β5i selectivity have been identified [41,42].

**Figure 8:**
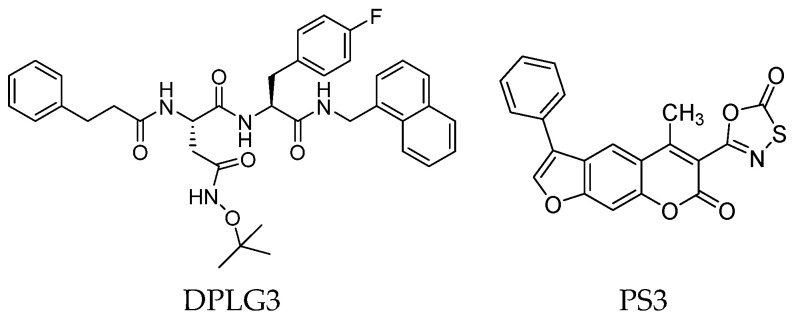
Two-dimensional structures of non-covalent immunoproteasome inhibitors DPLG3 and PS3.

Although non-covalent peptidyl compounds lack an electrophilic component at the *C*-terminus and thus cannot covalently bind to the catalytic Thr residue, some of these compounds maintain significant proteasome-inhibitory activity through their appropriate peptide backbone and side chains. For instance, the *N*,*C*-capped dipeptide DPLG3 (Figure 8) has been reported as a selective β5i inhibitor, targeting the subunit β5i with an IC_50_ value of 0.0045 μM and a remarkable 7200-fold greater selectivity than β5c [43]. However, due to its poor physicochemical properties and limited cell penetration, recent efforts have focused on improving the DPLG3 profile, resulting in the development of several derivatives that will be described in detail in Section 3. DPLG3 and its congeners are described as “active site-directed non-competitive inhibitors”, meaning their inhibition potency is not affected by increasing substrate concentrations. Nevertheless, in view of their peptide or peptidomimetic structure and their fitting into natural β5 substrate-binding sites [44], the validity of a non-competitive mode of action remains debatable.

As we have seen, most inhibitors have a peptide or peptide-like structure. In contrast, a very small number of non-peptide iCP inhibitors are reported in the literature. Among these, a variety of chemotypes, including metal complexes [45], have been described. In the search for a new potential non-peptidyl platform for designing alternative iCP inhibitors, computational techniques have proven to be valuable tools. For example, a computational docking approach led to the identification of the class of psoralenes as potential non-covalent iCP inhibitors. Out of them, the non-peptidic compound PS3 (Figure 8) acts on β5i with 107-fold greater selectivity than β5c (β5i, IC_50_ = 1.6 μM; β5c, IC_50_ = 172.2 μM) [46].

### 2.3. Immunoproteasome Inhibitors Targeting Non-Catalytic Residues

The usual covalent mode of action of β5i inhibitors often leads to undesirable biological activities and impairment of subunit selectivity, as all β subunits share very similar active site structures. More recently, the concept of non-catalytic residue targeting has been applied to the development of novel β5i inhibitors. Several independent studies have identified Cys48 in β5i, at the interface between the S2 and S4 pockets, as an isoform-specific nucleophilic residue [47,48]. This strategy aimed to achieve strong covalent blockage of β5i without affecting β5c subunit activity.

**Figure 9:**
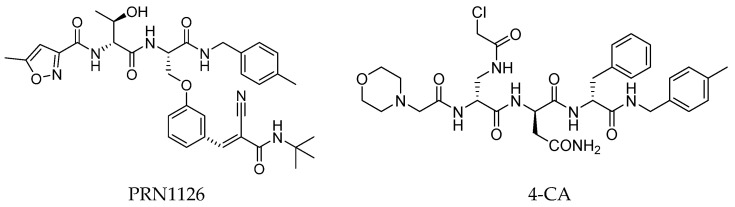
Chemical structures of inhibitors targeting the non-catalytic Cys48 residue 4-CA.

In 2015, Principia Biopharma developed PRN1126 and other covalent and reversible iCP inhibitors by introducing a nitrile moiety at the P2 position to link Cys48 residues (Figure 9) [49]. Although experimental studies demonstrating the reaction mechanism of PRN1126 are not available yet, it is thought that nucleophilic attack to the nitrile bond by the thiol likely results in a covalent thioimidate linkage [50].

Another representative of this unique class of covalent iCP inhibitors is the decarboxylated peptide 4-CA (Figure 9), which interacts with the non-catalytic Cys48 through an α-chloroacetamide warhead while its *C*-terminus has no additional group capable of undergoing nucleophilic addition with the catalytic Thr1. 4-CA exhibited up to 150-fold greater selectivity for β5i over β5c (β5i, IC_50_ = 2765 ± 255 nM; β5c, IC_50_ = > 10,000) [47].

## 3. SAR Studies of Immunoproteasome Inhibitors

In light of the reported side effects caused by indiscriminate inhibition of both cCP and iCP, the design of β5i-selective inhibitors is highly desirable. Extensive SAR studies have been conducted on various categories of β5i inhibitors, providing insights into the key molecular motifs needed to achieve selectivity. Immunoproteasome inhibitors can be classified into two major categories: peptide backbone-based inhibitors, the largest class, and non-peptide inhibitors (either natural or synthetic). This review aims to summarize the efforts made over the last five years in the design of selective β5i inhibitors with anti-cancer activity. The findings presented here might provide valuable insights into the structural requirements for selective immunoproteasome targeting.

### 3.1. Peptide Backbone-Based Immunoproteasome Inhibitors

**Figure 10:**
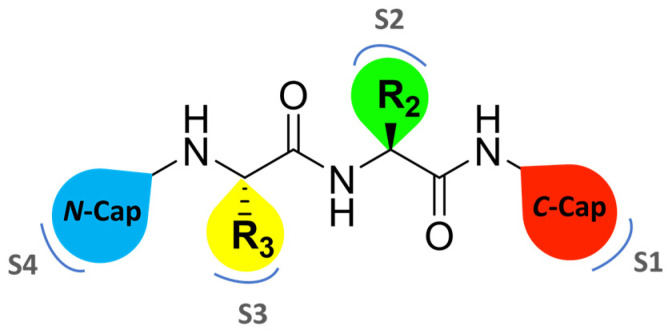
General structure of *N*,*C*-capped dipeptide lined to β5-binding sites (S1, S2, S3 and S4).

Zhan et al. recently published an extensive structure–activity relationship (SAR) study on a novel class of non-covalent proteasome inhibitors based on an *N*,*C*-capped dipeptide structure. Previous studies conducted by the same group led to the identification of the highly β5i-selective immunoproteasome inhibitor DPLG3 (see Section 2.2.2), which, unfortunately, was characterized by poor pharmacokinetic properties. In an effort to improve both the potency and physicochemical properties of this compound, they explored the *N*-cap (S4), R3 (S3), R2 (S2) and *C*-cap (S1) positions of the dipeptides (Figure 10) [51].

Preliminary investigations focused on the R2 group showed that replacing the *p*-F-phenylalanine in DPLG3 with *O*-methyl-serine, as in 1, or with alanine, as in 4, maintained potency against β5i but reduced isoform selectivity. This early SAR analysis suggested that modifications to the R2 group could be used to modulate both the physicochemical properties and the selectivity profile of the *N*,*C*-capped dipeptides.

Structural studies on iCP and cCP have shown that the S1 site governs the selectivity of inhibitors against β5i over the constitutive β5c [34]. The *C*-cap group binds to the S1 cleft, which is located close to the active site, thus becoming a major determinant in the regulation of β5i selectivity. Several attempts at *C*-cap optimization have been made, demonstrating that the naphtalenemethyl ring (R_1_) can drive selectivity toward β5i. Indeed, replacing the naphtalenemethyl moiety (R_1_) of **1** with a basic group such as 8-tetrahyquinolinmethyl (**2**) or 8-quinolinmethyl (**3**) significantly impairs interaction with the S1 hydrophobic pocket of β5i. Exploration of the *N*-cap (R_4_) revealed that appropriate hydrophobic *N*-cap groups can help tune selectivity. For instance, moving from the phenylpropionate group of **4** to the phenylbutanoate one on the *N*-cap of **5**, a significant increase in selectivity is observed (selectivity index from 230 to >5076) with a moderate decrease in β5i-inhibitory potency. A similar behavior was observed when switching to the phenylacetate derivative **6**, which showed nearly 10-fold greater selectivity compared to **4**. In contrast, introduction of less hydrophobic *N*-cap groups (e.g., pyrazine, compound **7**) led to a significant reduction in β5i-inhibitory activity and a dramatic loss in selectivity. Regarding the S_3_ region, substitution of Asn-*O*tBu with smaller and more polar groups, such as *O*-methyl-Ser (**8**) or unsubstituted Asn (**9**), abolished inhibitory activities against both β5i and β5c when compared to their parent compounds **4** and **1**, respectively. On the other hand, when replacing the Asn-*O*tBu in **DPLG3** with a Asn-neopentyl moiety as in compound **10**, the affinities for the two isoforms tend to converge, compromising the selectivity profile (Table 1). These results suggest that the presence, at R_3_, of an Asn substituted with a bulky aliphatic chain could improve β5i activity and offer a lever for selectivity tuning. To optimize the physicochemical properties of compound **1**, the authors synthesized its amide derivative by removing the O atom from the Asn-*O*tBu moiety, thus obtaining compound **11,** which showed a similar potency and selectivity profile but improved physicochemical features. Elongating the Asn-tBu chain of **11** to yield the Gln-*t*Bu derivative **12** resulted in similar β5i potency (β5i, IC_50_ = 0.0023 μM). This trend was also observed when replacing the R_3_ Asn-*O*tBu of **4** (β5i, IC_50_ = 0.0057 μM) with Gln-tBu (**13**, β5i, IC_50_ = 0.0015 μM), which increased β5i potency by 3.8-fold. However, potency towards β5c was enhanced as well, compromising β5i selectivity by 2-3-fold (**13** vs. **4**, **12** vs. **1**) (Table 1). Therefore, the authors selected Asn-*t*Bu (for selectivity) or Gln-*t*Bu (for potency) as the best R_3_ groups for further investigation. To increase the metabolic stability of **13**, the authors introduced a fluorine atom at position 4 of the naphtalenemethyl *C*-cap group, thus obtaining compound **14**, which showed potency comparable to that of the unsubstituted **13** but with a 2-fold improvement in β5i selectivity.

Based on the collected SAR information, they developed a hybrid compound (**15**) bearing both the R_3_ Asn-*t*Bu and the 4-F naphtalenemethyl group. This hybrid compound exhibited a substantial decrease in activity against β5i but a modest improvement in isoform selectivity (β5c/β5i = 204) compared to the Gln-*Ot*Bu analog **13**. The substitution of the phenylpropionate moiety of **14** and **15** with the 5-methylisoxazole-3-carboxylate group yielded compounds **16** and **17**, respectively, which showed improved β5i selectivity compared to their parent compounds (Table 1). An even more beneficial result was obtained by introducing a sulfonyl *N*-cap on Asn and Gln derivatives. Substitution of the phenylpropionate group in **13** with a tosyl one resulted in compound **18**, which showed IC_50_ values of 400 pM against β5i and 424 nM against β5c, yielding a selectivity of up to three orders of magnitude. Again, the introduction of a fluorine substituent on the naphtalenemethyl group of **18**, to give **19**, led to an improvement in β5i selectivity, although this was accompanied by a reduction in potency against β5i. The latter is affected even more negatively when the R_2_ Gln-*t*Bu is replaced by Asn-*t*Bu (**20**). This trend was confirmed with the R_2_ *O*-methyl-serine derivatives **21** and **22**, where the Gln-*t*Bu **21** was 70-fold more active than the Asn-*t*Bu derivative **22** (Table 1). Compound **21** also showed the best result in inhibiting T-cell proliferation in peripheral blood mononuclear cells (PBMCs) upon stimulation by the anti-CD3 antibody. This extensive SAR study highlighted several factors that significantly influence the potency and selectivity of *N*,*C*-capped dipeptides: (1) the presence of a bulky and hydrophobic *C*-cap (P1) is the primary determinant of selectivity against β5i over β5c; (2) a glutamine with a tert-butyl amide side chain (Gln-*t*Bu, P3) strongly improves potency (by 3- to 50-fold) against β5i compared to P3 Asn, without influencing the selectivity; and (3) a hydrophobic *N*-cap is preferred for optimizing selectivity.

**Figure 11:**
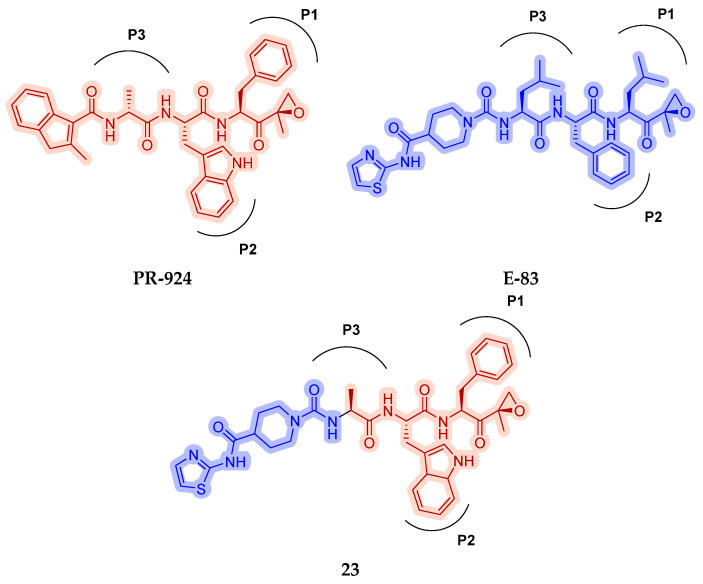
Chemical structures of immunoproteasome inhibitors PR-924 and E-83 and the corresponding hybrid compound **23** [52].

As mentioned above, ONX-0914 (β5i, IC_50_ = 39 nM; β5c/β5i ratio: 7.0) was the first immunoproteasome-specific inhibitor targeting the highly active β5i subunit (Table 2). Over time, it emerged as a key compound for iCP inhibitor development. PR-924 is a β5i-selective tripeptide epoxyketone proteasome inhibitor which covalently binds to the proteasomal *N*-terminal threonine. The chemical structures of ONX 0914 and PR-924 differ in their P2 residue (methoxy tyrosine vs. tryptophane), in the stereochemistry of their P3-Ala residue (L vs. D) and in their *N*-cap (morpholine versus 3-methyl-1*H*-indene; Figure 7). Recently, the Li research group reported the discovery of a novel series of β5i-specific inhibitors though a fragment-based drug design approach [52]. They initially proposed a hybrid compound (**23**) combining the P1, P2 and P3 moieties of PR-924 with the *N*-Cap group of compound E-83, a potent proteasome inhibitor identified by the same group (Figure 11). Compound **23** exhibited stronger β5i-inhibitory potency (β5i, IC_50_ = 21.1 ± 0.3 nM) compared to the positive control ONX-0914 (Table 2). The authors speculated that this slight improvement could be due to the simultaneous presence of the bulky phenyl group at R_1_ and of the 3-indolyl moiety at R_2_. Preliminary SAR studies revealed differences in potency and selectivity between L- and D-configuration derivatives (**23** vs. **24**). Compounds with L-Ala at the R_3_ position were more potent, while those with D-Ala were more selective. Molecular docking simulations suggested that the *N*-Cap group in the D-Ala derivative (**24**) caused a steric clash within the β5i S3 pocket, likely due to the length of the 4-(thiazol-2-yl)-piperidine *N*-cap. These clashes were instead absent when L-Ala was present in R_3_ (**23**). Based on these findings, the authors designed a second series of compounds featuring a shorter *N*-cap group, retaining the phenyl moiety at P1, introducing a 4-methoxyphenyl group at P2 and using either D-Ala or L-Ala at P3. This led to the development of a novel library of ten ONX-0914 analogs. Among these, derivatives **25** (β5i, IC_50_ = 44.5 ± 0.7 nM; β5c/β5i ratio: 24.8) and **26** (β5i, IC_50_ = 26.0 ± 6.28 nM; β5c/β5i ratio: 24.9), both bearing P3–D-Ala, were the most potent and selective β5i inhibitors (Table 2). The findings highlighted the importance of less bulky *N*-cap groups (e.g., 4-methoxypiperidine, **26**; 3-methoxy-pyrrolidine, **25**) for optimal β5i binding. However, compound **26** demonstrated only moderate anti-proliferative activity against RPMI-8226 and MM.1S cell lines, suggesting that inhibition of β5i alone is not always sufficient to exert anti-cancer activity.

**Figure 12:**
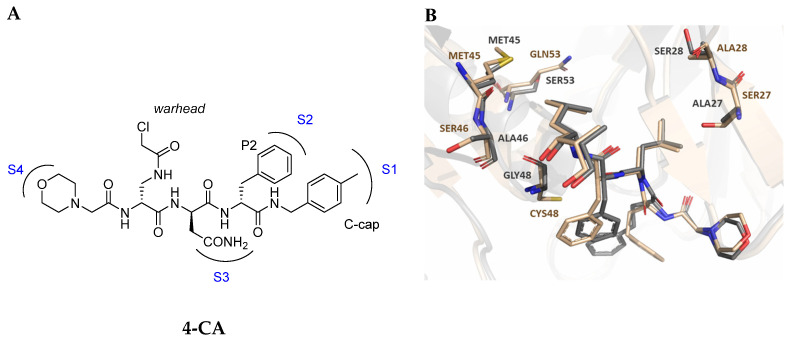
(**A**) Molecular structure of 4-CA and 2D interaction diagram within β5i; (**B**) superimposition between yeast 20S proteasome with human β5i (1–138) and human β6 (97–111; 118–133) in complex with carfilzomib (PDB ID: 5L5E, wheat color) and yeast 20S proteasome with human β5c (1–138) and human β6 (97–111; 118–133) in complex with carfilzomib (PDB ID: 5L5Y, dark gray color).

As previously mentioned, most of the known iCP inhibitors feature a peptide backbone bearing an electrophile *C*-cap that interacts with the active site residue Thr1. Due to the similar binding modes within the active sites of β5i and β5c, these inhibitors often simultaneously affect the constitutive cCP. However, as described in Section 2.3, a decarboxylated tripeptide, named 4-CA (β5i, IC_50_ = 2765 ± 255 nM; β5c, IC_50_ = > 10,000, Figure 12A), has been identified as a selective inhibitor. It covalently targets a non-catalytic Cys residue of the β5i subunit through an α-chloroacetamide-containing sidechain, achieving a 150-fold greater selectivity for β5i over β5c [47]. The singular interaction mode of 4-CA has positioned it as a promising chemical template for the development of novel immunoproteasome-selective inhibitors. Building on the structure of 4-CA, the Nan research group designed a novel series of forty-eight *N*,*C*-capped di- and tripeptides. The rationale for the design of the 4-CA derivatives stemmed from the analysis of the X-ray crystal structures of carfilzomib bound to β5i (PDB ID: 5L5E) and β5c (PDB ID: 5L5Y) in a chimeric yeast/human proteasome (Figure 12B) [53]. This structural analysis revealed a key residue inversion, in the S3 pocket, between β5i and β5c (Ser27Ala28 for β5i, and Ala27Ser28 for β5c), thus providing the authors with a rationale for the modification of P3 moieties. In this analysis, longer linkers with hydrophobic tails, headed toward the S3 pocket, were expected to promote the binding to β5i. Hence, the authors began the SAR exploration by changing the P3 from the Asn of 4-CA to groups with bulkier tails.

Compounds with larger hydrophobic tails demonstrated improved potency against β5i, likely due to a better fit within the wider surface area of the β5i S3 pocket. Among these, compound **27** (β5i, IC_50_ = 3.54 ± 0.09 nM; β5c, IC_50_ = 50.78 ± 4.05 nM; β5c/β5i ratio: 14) was the most potent derivative, with an IC_50_ against β5i three orders of magnitude greater than that of 4-CA, and this made it a candidate for further optimization of the *N*-cap region (targeting the S4 pocket). However, replacing the *N*-cap morpholine ring of compound **27** with several fragments (e.g., phenylbutanoate, 3-indoleglyoxylate, tosyl) or even removing it (compound **28**; β5i, IC_50_ = 3.90 ± 0.11 nM; β5c, IC_50_ = 48.21 ± 2.86 nM; β5c/β5i ratio: 12) did not lead to any significant improvement in terms of activity or selectivity index. These results suggest that the β5i subunit is not particularly sensitive to structural changes on the *N*-cap (Table 3).

Conversely, differences in the S1 pockets between β5i and β5c, such as the orientation of Met45 in β5i (Figure 12B), creating a wider pocket, play a critical role in isoform selectivity [34]. Indeed, bulkier P1 groups might be better accommodated within the β5i S1 pocket, driving selectivity. Based on this assumption, the authors explored various *C*-cap groups with different steric and electronic effects, starting from compound **28**. The 1-naphtalenmethyl moiety emerged as an optimal *C*-cap group (compound **29**; β5i, IC_50_ = 3.82 ± 0.44 nM; β5c, IC_50_ = 431 ± 76 nM; β5c/β5i ratio: 113), supporting earlier observations by Zhan et al. [51].

For the S2 pocket, variations in the substitution pattern of the phenyl ring (electron-donating or electron-withdrawing) generally resulted in slightly less active compounds. An exception was found for compound **30** (β5i, IC_50_ = 3.81 ± 0.22 nM; β5c, IC_50_ = 230 ± 24 nM; β5c/β5i ratio: 60), which featured a 2-naphtalene group at R2 and displayed the highest potency and selectivity in this subseries. Conversely, the reduction in the R_2_ steric hinderance from benzyl (**28**) to smaller groups (e.g., methoxy, methyl, hydrogen) led to decreased activity against both β5i and β5c, presumably due to suboptimal occupation of the S2 pocket.

To address concerns about irreversible protein modification, the α-chloroacetamide warhead (covalently binding to the β5i-specific Cys48) was replaced with a 2-cyanoacrylamide moiety, a typical covalent but reversible warhead. However, modifications at the 3-position of the 2-cyanoacrylates did not significantly affect β5i-inhibitory activity. Among the acrylate derivatives, only the 4-chlorophenyl-substituted one (compound **31**; β5i, IC_50_ = 3.67 ± 0.42 nM; β5c, IC_50_ = 80.61 ± 3.23 nM; β5c/β5i ratio: 22) showed modestly improved β5i selectivity compared to compound **28**. Incorporating the optimal 1-naphtalene R_1_ group into compound **31** yielded derivate **32** (β5i, IC_50_ = 8.75 ± 1.24 nM; β5c, IC_50_ = 1087 ± 218 nM; β5c/β5i ratio: 124), with a 6-fold increase in β5i/β5c selectivity. Introducing a 2-naphatelene fragment at R_2_ further enhanced selectivity and slightly increased in β5i potency (compound **33**; β5i, IC_50_ = 1.74 ± 0.15 nM; β5c, IC_50_ = 387 ± 34 nM; β5c/β5i ratio: 222). Surprisingly, reverting to the covalent α-chloroacetamide warhead resulted in compound **34** (β5i, IC_50_ = 1.07 ± 0.23 nM; β5c, IC_50_ = 572 ± 57 nM; β5c/β5i ratio: 535), which showed significantly improved selectivity, suggesting that a different interaction mode may be involved in the inhibition mechanism. The key role of electrophile warheads in both β5i inhibition and selectivity was further confirmed through the replacement with unreactive analogs (e.g., compound **35**), which had detrimental results (Table 3).

The extensive SAR exploration on 4-CA conducted by Nan et al. provided novel *N*,*C*-capped di- and tripeptides with nanomolar activity against β5i and β5i selectivity values with a range across two orders of magnitude [53]. Some of the identified peptides also demonstrated significant cell growth inhibition against a human colorectal carcinoma cell line (HTC-116), with IC_50_ values ranging from 0.16 to 1.70 µM. These results were comparable to or even better than the reference compound ONX0914 (β5i, IC_50_ = 0.73 ± 0.10 µM).

### 3.2. Non-Peptide Backbone-Based Immunoproteasome Inhibitors

Schiffrer et al. focused their SAR studies on psoralen derivatives as β5i inhibitors. In previous work, they converted non-covalent inhibitors into covalent ones by introducing an electrophilic warhead at position 3 of the psolaren ring, obtaining compounds with selectivity against the β2i and β1i subunits [54]. Among these, the derivative PS3, which features an oxathiazolone warhead and an aromatic ring at position 4′, demonstrated the highest selectivity for the β5i subunit (Figure 8) [46].

**Figure 13:**
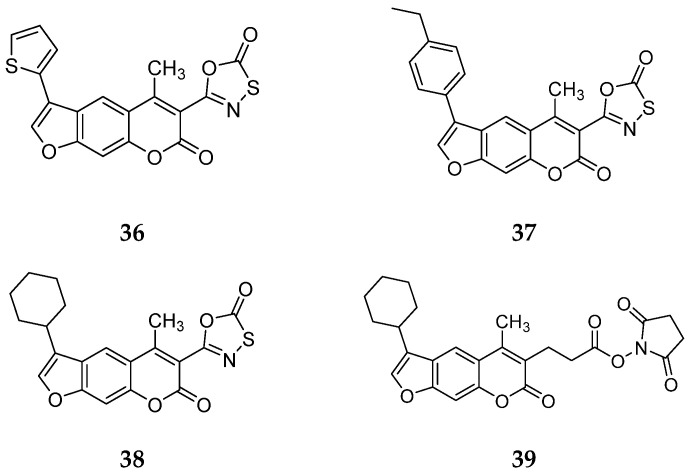
Chemical structures of the psolaren derivates **36**–**39**.

In this work, Schriffrer et al. synthesized a new series of psolaren derivatives, retaining the oxathiazolone at position 3 while introducing some different substituents at position 4′, to assess their effects on activity and selectivity. PS3 was modified at position 3 to evaluate the impact of different electrophilic warheads. At position 4′, they introduced variously substituted phenyl rings (in *ortho*, *meta* and *para* positions), bioisosteres and cycloalkyl rings. All compounds were tested in fluorimetric assays using succinyl-Leu-Leu-Val-Tyr-AMC as the fluorogenic substrate, exhibiting submicromolar activity against the β5i subunit. The most active compounds included those bearing a thiophene (**36**), a para ethyl phenyl (**37**) and a cyclohexyl (**38**) at position 4′, with β5i IC_50_ values of 141 nM, 174 nM and 106 nM, respectively (Figure 13). Regarding derivatives bearing substituents in the *para* position, the presence of bromine or fluorine led to a reduced activity, as did the introduction of *ortho*- or *meta*-methoxy or *meta*-methyl groups. However, activity improved with the introduction of larger cycloalkyl groups, as observed for compound **38**.

To further investigate the effect of the electrophilic group on inhibition properties, the oxathiazolone at position 3 of compound **38**, the most promising derivate, was replaced with alternative ones. The introduction of a nitrile group determined loss of activity, whereas only the succinimidyl-ester **39** showed moderate activity (IC_50_ = 1830 nM) (Figure 13).

All compounds were also tested against the constitutive proteasome to assess their selectivity. Although several IC_50_ values could not be determined due to solubility issues, compounds **36**–**38** demonstrated 100-fold greater selectivity, marking a good improvement over PS3 [46].

To evaluate the immunoproteasome inhibition properties in the presence of other cytosolic components, compounds **37** and **38** were tested against HeLa and THP-1 cells. Both compounds exhibited enhanced activity against THP-1 cells, with IC_50_ values of 1.53 µM for compound **37** and 1.01 µM for compound **38**.

The development of non-peptide immunoproteasome inhibitors remains a challenging task, but in spite of cell permeability issues, the described psolaren derivatives provided a promising starting point for the development of new derivatives.

By comparing psoralen derivatives with structural modification at the 4′ position and different electrophilic warheads at position 3, compound **36**—bearing an oxathiazolone ring at position 3—emerged as the most promising [46]. It was demonstrated that the oxathiazolone ring binds to Thr1 at the active site through a cyclocarbonylation reaction involving β-OH and α-NH_2_.

**Figure 14:**
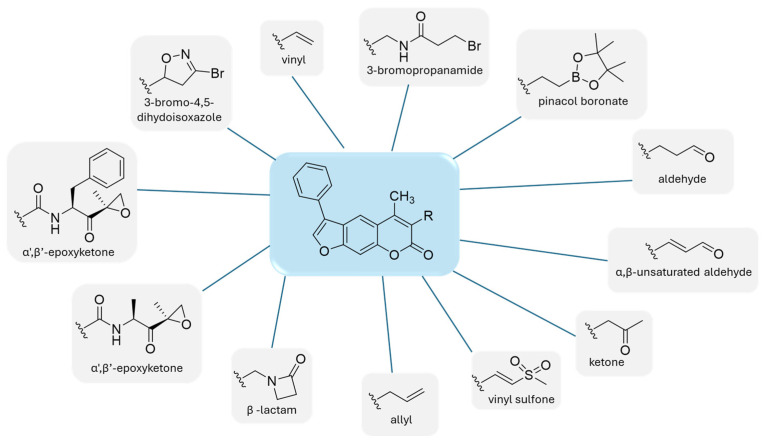
Warheads introduced in the psolaren inhibitors.

Following observations that substituting the oxathiazolone group at position 3 with warheads such as nitrile and acrylamide led to reduced β5i inhibition, Schiffrer et al. further explored this position using alternative groups, as shown in Figure 14. The selected warheads were chosen based on their potential reactivity with the active site Thr residue. All compounds were synthesized and tested against all subunits of both the immunoproteasome and the constitutive proteasome, using various fluorogenic substrates [55].

**Figure 15:**
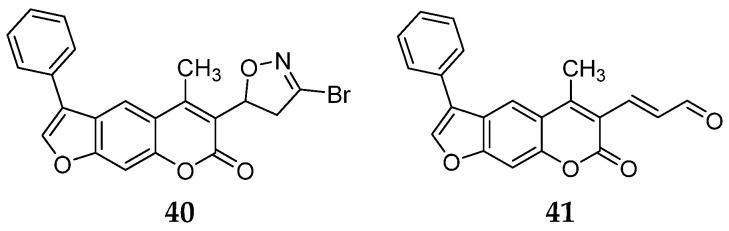
Psolaren inhibitors **40**–**41**.

When comparing the results of compound **36** with those of carfilzomib, its activity against all six tested subunits was relatively poor. All compounds exhibited similar potency against the β5i subunit, with residual activity (RA) ranging from 62% to 78%. Among the 12 synthesized compounds, compound **40,** featuring a 3-bromo-4,5-dihydoisoxazole, and compound **41,** containing an α,β-unsaturated aldehyde, demonstrated the most favorable profiles (Figure 15).

The low activity observed for compounds bearing the above mentioned electrophilic warheads may be attributed to a suboptimal distance between the electrophilic carbon of the inhibitor and the Thr Oγ in the active site-bound conformation, which does not allow the formation of a covalent bond.

**Figure 16:**
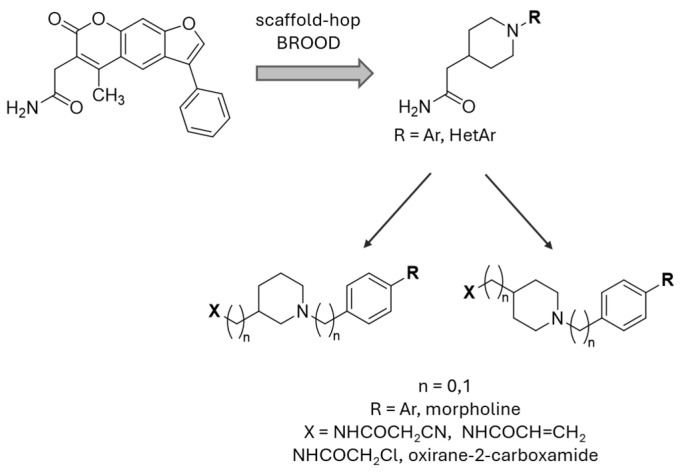
Computational design of the new 3- or 4-substituted amino- and aminomethyl piperidine derivates.

A ligand-based computational study for the design of non-peptide immunoproteasome inhibitors was reported in 2021 by Gobec et al. [56]. A fragment library was created using CheMBL 20, the Drugbank database and CHOMP. BROOD/vBROOD was then used to perform, from the starting psoralene-based parent structure, a scaffold hopping in silico procedure that finally identified 3- or 4-substituted amino- and aminomethyl piperidine scaffolds as the highest-ranking hits (Figure 16).

The synthetic feasibility of the in silico identified scaffolds was evaluated, considering the introduction of different electrophilic warheads at position 3 and 4 of the piperidine ring. The introduction of the electrophilic warheads was guided by the results from previous studies [46,54,55].

All synthetized compounds were tested in fluorimetric assays against the β5i subunit. Compounds with good solubility in the assay buffer and an inhibition percentage above 20% were further characterized (Table 4).

Those bearing an acrylamide warhead exhibited IC_50_ values in the low micromolar range. The methylene group linking the phenyl ring to the piperidine one was found to be crucial for activity, as only compounds **42**–**44** displayed significant inhibition of the β5i subunit. These compounds were also tested against the β5c subunit, revealing comparable potency against cCP and iCP (Table 4). Compound **44** emerged as the most potent inhibitor of both the constitutive proteasome and immunoproteasome.

**Figure 17:**
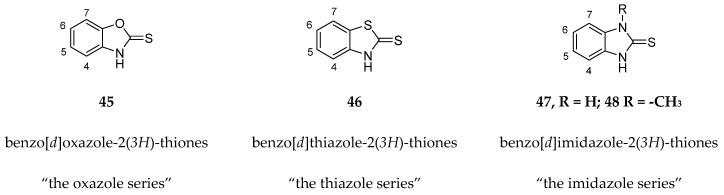
Structures of parent compounds **45**–**48** and their derivates.

Kollar et al. identified benzoxazoles-2(*3H*)-thiones as inhibitors of chymotrypsine-like activity of the immunoproteasome from the screening of an in-house library of fragments. Based on IC_50_ values and synthetic accessibility, seven hits were selected for further studies. Therefore, 61 commercially available and structurally related compounds were screened against β5i, leading to the identification of four different subseries, whose parent compounds **45**–**48** are shown in Figure 17 [57].

To evaluate the impact of structural modifications on inhibitory activity, various substituents of different nature (-Cl, -OH, -OCH_3_, -CH_3_, -COOCH_3_, -NO_2_) were introduced at positions 4, 5, 6 and 7 of the four parent compounds. The presence of a chlorine atom at 6- and 7-positions of benzoimidazole derivatives improved activity compared to parent compounds (compounds **49** and **50**, Table 5), while in the case of benzothiazole and benzoisoxazole derivatives, it resulted in reduced or similar activity.

The introduction of a hydroxyl group at position 4 enhanced activity in the oxazole series only, with compound **51** exhibiting an IC_50_ of 4.1 ± 0.9 µM (Table 5). Regarding position 5, the best result was observed with compound **52**, a thiazole derivative with a methoxy substituent (IC_50_ = 7.2 ± 6.1 µM). At position 6, different substituents were introduced across oxazole, thiazole and imidazole derivatives, with compound **53** (COOCH_3_ substitution) showing the most promising activity (Table 5).

Given the presence of an acidic cysteine in the active site but not in the catalytic region, the potential for disulfide bond formation with Cys48 was considered. Consequently, new 1,3-benzothiazole derivatives were synthesized with several warheads, including vinylsulfone, 2-sulfonyl-fluoride, 2-acrylamide and 2-nitrile groups. Only derivatives **54** and **55** (Table 6) exhibited notable activity against β5i. Compound **55**, featuring a 2-sulfonyl-fluoride warhead, demonstrated the highest β5i-inhibitory activity (IC_50_ = 11 ± 2.0 µM). However, compound **54**, with a 2-nitrile warhead, although exhibiting a higher IC_50_ (83 ± 6.0 µM), was reported to be the most selective and therefore the most promising compound.

Further modifications were made to benzoxazoles-2-carbonitriles, specifically by introducing chlorine atoms at positions 6 and 7, as these substitutions were previously shown to improve activity, and as expected, compounds **56** and **57** exhibited improved activity over compound **54** (Table 6).

Building on this work, Kollar et al. focused on developing novel benzoxazole-2-carbonitriles, starting from compound **52** [57]. While their previous study primarily assessed the influence of chlorine at positions 6 and 7 [57], this research explored the impact of lipophilic and hydrogen-bonding substituents (-CH_3_, -OH, -OCH_3_, -OCOCH_3_, -NO_2_, -NH_2_, -NHCOPh) at various positions. Stability in assay buffer and β5i-inhibitory activity were evaluated. Compounds **58**–**60**, featuring hydroxyl groups at positions 4, 5 and 7, displayed the highest activity, with IC_50_ values ranging from 2.1 µM to 4.2 µM. Compound **61**, containing an -OCOCH_3_ group at position 5, exhibited similar potency (Table 7). All these compounds were stable in the assay buffer [48].

The selectivity was assessed through fluorimetric assays against the constitutive proteasome and β1i and β2i. Despite the presence of some inhibitory activity against β5, the benzoxazole-2-carbonitriles demonstrated fair selectivity.

**Figure 18:**
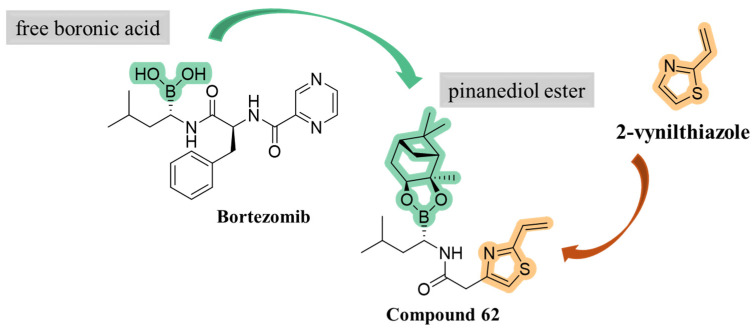
Development of compound **62**.

To identify novel heterocyclic electrophilic fragments by library screening, 2-vinylthiazole was selected to be introduced into a potential covalent inhibitor, in addition to a bortezomib-inspired warhead. Compound **62** was synthesized as pinanediol ester instead of free boronic acid for synthetic reasons but pinanediol ester derivatives were shown to have comparable potency to boronic acid ones (Figure 18) [58,59,60,61].

**Figure 19:**
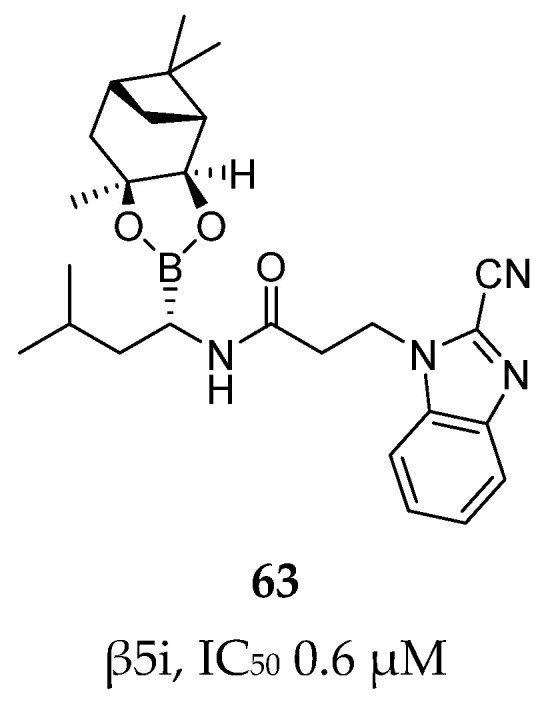
Compound **63**: chemical structure and inhibitory activity against the β5i subunit.

Using the same strategy, Kollar et al. synthesized a new series of compounds linking carbonitrile heterocycle (benzoxazole and benzimidazole) to an (*R*)-boroleucine moiety via various linkers. The most potent inhibitor, compound **63** (Figure 19), featured an ethylene linker and had an IC_50_ value of 0.6 µM. Contrary to what had been observed in previous studies, introduction of a chlorine atom did not enhance β5i-inhibitory activity in this case.

The bidentate series was further evaluated against the constitutive proteasome and β1i and β2i to assess selectivity. While none of the compounds exhibited activity against β2i and β2c, they retained low to submicromolar potency against the other proteasome subunits. Compound **63** exhibited the following IC_50_ values: 1.2 ± 0.1 µM for β1i, 0.4 µM for β1c and 0.7 ± 0.1 µM for β5c.

These findings suggest that the two most potent benzoxazole-2-carbonitrile inhibitors, i.e., compound **58** and **60**, are promising candidates for future optimization and development of novel selective non-peptide inhibitors. Regarding the bidentate series, these compounds may serve as a starting point for the design of new derivatives targeting multiple proteasome subunits.

Klein et al. initially investigated the pharmacological profiles of commercially available proteasome inhibitors (bortezomib, ixazomib and carfilzomib) (Figure 3). Their activity was evaluated against both the constitutive proteasome and immunoproteasome using subunit-specific fluorogenic substrates. The results, summarized in Table 8, indicated that the inhibitors were active against both proteasome isoforms, with carfilzomib exhibiting the lowest selectivity [62].

**Figure 20:**
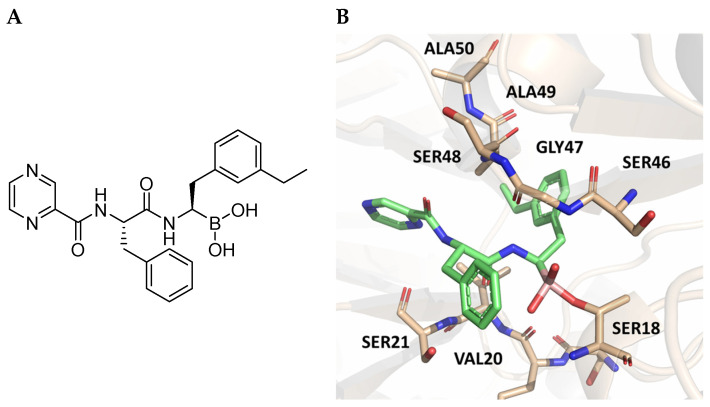
(**A**) Chemical structure of compound **64**; (**B**) co-crystal of compound **64** with the human immunoproteasome (PBD ID: 7B12).

The authors resolved a co-crystal structure of the human immunoproteasome in complex with compound **64** (Figure 20), a bortezomib derivative they synthesized in which the isobutyl group was replaced with a 3-ethyl-benzyl group (Figure 20, Table 8). This modification enhanced selectivity, with a β5i/β5c selectivity index of 24. The boron atom binds to Thr1, forming a coordinated covalent tetrahedral adduct. In the β5i subunit, the 3-ethylbenzyl group, bulkier than the isobutyl of bortezomib, fits well in the S1 pocket (Figure 20). Compound **64** retains the same dipeptide sequence as bortezomib, forming strong H-bonds with Ser21 and Gly 47 near the active site, as well as with Ser21 and Ala49 in β5i. Similar interactions were observed in β5c.

**Figure 21:**
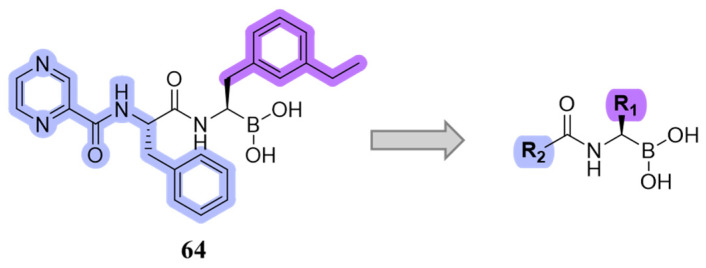
Design of new boronate derivates starting from compound **64**.

Starting from compound **64**, SAR studies were carried out to investigate the selectivity of inhibitors. In particular, R_2_ was replaced with sulfonamides or tertiary amides, but these derivates did not exhibit good pharmacokinetic properties. For this reason, R_2_ was replaced with groups that have few H-bond acceptors or donors. R_1_ was replaced with bulky substituents to better fit into the S1 pocket (Figure 21).

The new boronate derivatives, in which different R groups were introduced, were synthesized and tested to evaluate their activity and selectivity against the immunoproteasome. During the synthesis, the two diastereomers *R* and *S* were obtained and separated to determine the chiral purity and the influence of the different configurations on immunoproteasome inhibition. As previously reported by Zhu et al. [60], the authors confirmed that the *R* diastereomers were 10- and 100-fold more active than the *S* diastereomers against β5i.

The authors initiated SAR studies by investigating the S1 pocket and synthesized new derivatives with a benzyl group as R_2_ (Table 9). The presence of the methylene linker allowed a torsion of the benzyl group which interacted with Cys48 via van der Waals interactions. The benzyl group was introduced because, although it was not the most potent substituent, it was the most selective. The most active compounds from the first series were derivatives **65**–**67**, whose activity against β5i improved as the lipophilic character increased (compound **65** IC_50_ = 1000 ± 120 nM, compound **66** IC_50_ = 160 ± 340 nM, compound **67** IC_50_ = 85 ± 2.8 nM). Compound **67**, with a 2,4-dimethyl-benzyl as R_2_, was the most selective with a β5c/β5i ratio of 182.

In a second compound series, the authors introduced bicyclic aromatic systems to improve selectivity, obtaining the 3-benzo-furanyl derivative **68**, which was approximately 10-fold more potent than the previously synthesized derivatives (**65**–**67**) (IC_50_ = 2.1 ± 1.0 nM), while also enhancing selectivity. Compound **68** exhibited a good pharmacokinetic profile and stability in mouse and human liver microsomes. Clearence, volume of distribution and half-life time were also evaluated, yielding good results. Although benzofuran is known for its metabolic instability [63], the presence of the polar boronic acid group preserved it from oxidative metabolism. Small substituents at positions 2, 6 and 7 were introduced on the benzofuran ring to assess their influence on activity and selectivity. Compound **69**, bearing a methyl group on the benzofuran ring, was the most promising in terms of its selectivity with a β5c/β5i ratio of 2334.

After identifying the best R_1_ substituent, the authors proceeded with investigating the R_2_ group. The first series of the new derivatives retained the 3-benzo-furanyl group as R_1_ while replacing the benzyl group with *N*-containing mono- or bicyclic systems. These derivatives exhibited good activity towards the β5i subunit with an IC_50_ in the low nanomolar range but showed lower selectivity compared to previously synthesized compounds. Conversely, compounds bearing a phenylcianide (**70**–**73**) exhibited very good selectivity (Table 10).

**Figure 22:**
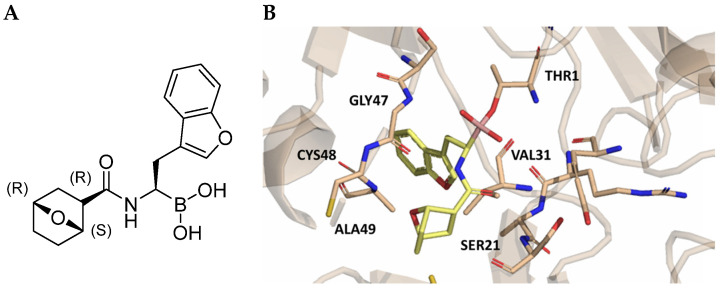
(**A**) Chemical structure of compound **76**; (**B**) co-crystal of compound **76** with the human immunoproteasome (PBD ID: 7AWE).

The introduction of H-bond acceptor groups such as α- or β-ethers led to compounds **74** and **75**, which demonstrated good potency and selectivity. The incorporation of an oxygen atom into mono- or bicarbocycles yielded compounds **76**–**78**. Given its good activity in terms of potency and selectivity in both enzyme and cellular assays (Table 10), compound **76** emerged and was extensively characterized. It was co-crystalized to evaluate its interaction with the catalytic site of the β5i subunit (Figure 22), and further investigation confirmed its metabolic stability.

Compound **76** was also evaluated in cell toxicity and in vivo pharmacokinetic studies in mice xenografted with U266B1 MM cells. Given the promising results, preclinical phase I studies have recently been conducted for the treatment of refractory MM.

## 4. Conclusions

Over the past decade, various chemical entities have been investigated for their anti-cancer potential through immunoproteasome inhibition. Based on their chemical structures, these compounds can be broadly classified into two categories: peptide-backbone-based inhibitors, the most common class, and non-peptidic derivatives (either natural or synthetic), which are less prevalent. Most reported peptidyl inhibitors function via a covalent inhibition mechanism, featuring an electrophilic warhead at the C-termini that covalently binds to the catalytic residue Thr1. However, this mode of action may often lead to adverse side effects and reduced subunit selectivity, as all β subunits share a very similar active site architecture. To address these challenges, several non-covalent immunoproteasome (iCP) inhibitors, including both peptidyl and non-peptidyl structures, have recently been identified. Among them, the non-covalent *N*,*C*-capped dipeptide DPLG3 and its analogs have demonstrated remarkable improvements in potency and selectivity against β5i compared to currently approved inhibitors or those under clinical investigation, which typically exhibit broad-spectrum activity. While non-peptidyl iCP inhibitors reported to date have yet to achieve comparable inhibitory activity and selectivity to their peptidyl counterparts, their structural diversity presents opportunities for further optimization. This might be a promising avenue for developing novel immunoproteasome-selective inhibitors. In conclusion, despite the extensive repertoire of known iCP inhibitors—some of which are already approved for clinical use—there is still an unmet need for more effective, manageable and safe inhibitors. Additionally, the identification of more selective compounds could provide valuable tools for further elucidating proteasome functions, pathways and modulation. Literature analysis already provides much information from structure–activity relationship, biological, clinical and structural studies, which could significantly accelerate the discovery of novel, optimized immunoproteasome inhibitors as potential therapeutic options for the treatment of tumoral pathologies.

## Figures and Tables

**Table 1:** Chemical structures and inhibition IC_50_ values for compounds **1–22** against human iCP β5i and cCP β5c.

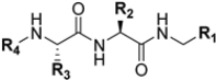							
Cmpd.	R_4_	R_3_	R_2_	R_1_	β5i IC_50_ (μM)	β5c IC_50_ (μM)	β5i Selectivity (β5c/β5i Ratio)
**DPLG3**	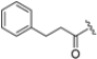	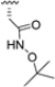	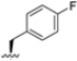	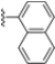	0.0045	32.4	7200
**1**	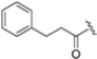	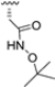	CH_3_OCH_2_-	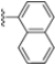	0.0044	0.3	68
**2**	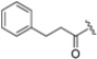	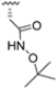	CH_3_OCH_2_-	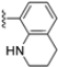	0.501 ± 0.328	27.23 ± 25.43	54
**3**	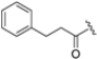	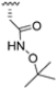	CH_3_OCH_2_-	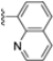	0.0837 ± 0.0302	1.04 ± 0.159	12
**4**	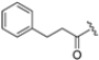	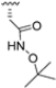	CH_3_-	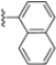	0.0057 ± 0.0018	1.32 ± 0.43	230
**5**	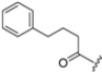	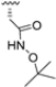	CH_3_-	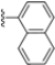	0.0197 ± 0.0011	>100	>5076
**6**	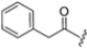	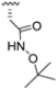	CH_3_-	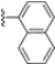	0.0259 ± 0.0024	47.67 ± 30.19	1840
**7**	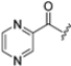	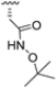	CH_3_-	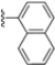	2.812 ± 0.512	>100	>35
**8**	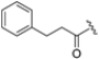	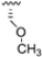	CH_3_-	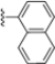	21.65 ± 10.99	>100	>5
**9**	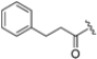	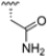	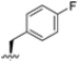	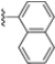	35.0 ± 18.5	>100	>2.8
**10**	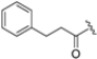	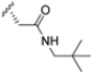	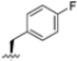	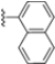	0.020 ± 0.05	0.307 ± 0.101	15
**11**	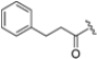	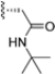	CH_3_OCH_2_-	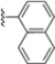	0.0072 ± 0.0029	0.514 ± 0.034	71
**12**	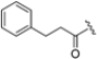	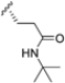	CH_3_OCH_2_-	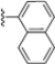	0.0023 ± 0.0011	0.055 ± 0.015	24
**13**	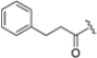	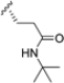	CH_3_-	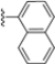	0.0015 ± 0.004	0.12 ± 0.023	80
**14**	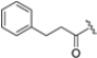	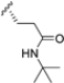	CH_3_-	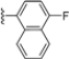	0.0016 ± 0.002	0.244 ± 0.021	152
**15**	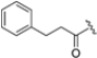	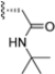	CH_3_-	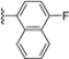	0.0508 ± 0.0053	10.73 ± 3.38	204
**16**	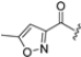	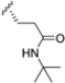	CH_3_-	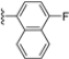	0.0012 ± 0.0004	0.61 ± 0.221	508
**17**	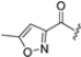	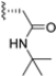	CH_3_-	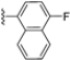	0.188 ± 0.0015	28.52 ± 2.88	984
**18**	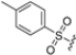	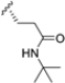	CH_3_-	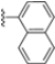	0.0004 ± 0.0002	0.424 ± 0.202	1208
**19**	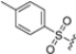	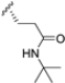	CH_3_-	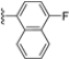	0.0024 ± 0.0003	5.52 ± 0.928	2329
**20**	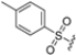	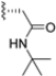	CH_3_-	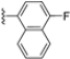	0.0164 ± 0.0022	53.7 ± 13.3	3274
**21**	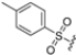	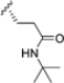	CH_3_OCH_2_-	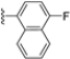	0.0008 ± 0.0003	1.456 ± 0.829	1881
**22**	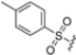	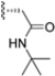	CH_3_OCH_2_-	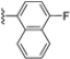	0.056 ± 0.002	13.74 ± 1.866	246

**Table 2:** Chemical structures and inhibition activity/selectivity profile for peptide epoxyketones ONX-0914 and **23–26 [52]**.

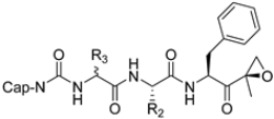						
Cmpd.	R_3_	*N*-Cap	R_2_	β5i IC_50_ (μM)	β5c IC_50_ (μM)	β5i Selectivity (β5c/β5i Ratio)
**ONX-0914**	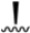	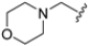	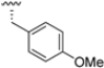	28.2 ± 9.2	196.7 ± 77.0	7.0
**23**	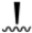	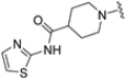	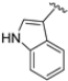	21.1 ± 0.3	25.6 ± 1.0	1.2
**24**	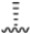	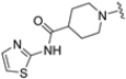		245.1 ± 117.5	468.2 ± 69.6	1.5
**25**	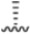	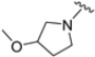	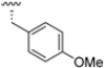	44.5 ± 0.7	1106.6 ± 338.6	24.8
**26**	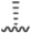	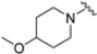		26.0 ± 6.28	647.1 ± 121.0	24.9

**Table 3:** Chemical structures and inhibition activity/selectivity profile for *N*,*C*-capped di- and tri-peptides 4-CA and **27**–**35** [53].

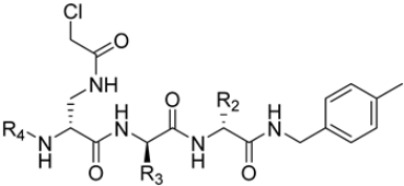						
Cmpd.	R_4_	R_3_	R_2_	β5i IC_50_ (nM)	β5c IC_50_ (nM)	β5i Selectivity (β5c/β5i Ratio)
**4-CA**	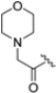	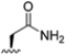	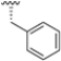	2765 ± 255	>10,000	>4
**27**	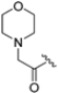	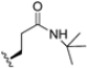	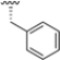	3.54 ± 0.09	50.78 ± 4.05	14
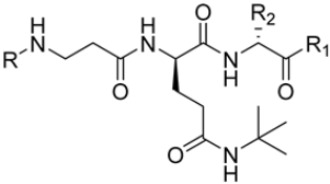						
**Cmpd.**	**R**	**R_2_**	**R_1_**	**β5i** **IC_50_ (nM)**	**β5c** **IC_50_ (nM)**	**β5i Selectivity** **(β5c/β5i Ratio)**
**28**	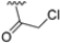	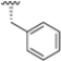	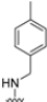	3.90 ± 0.11	48.21 ± 2.86	12
**29**	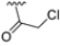	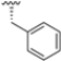	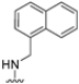	3.82 ± 0.44	431 ± 76	113
**30**	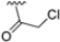	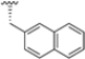	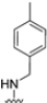	3.81 ± 0.22	230 ± 24	60
**31**	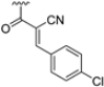	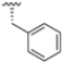	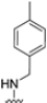	3.67 ± 0.42	80.61 ± 3.23	22
**32**	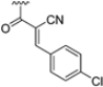	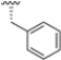	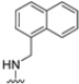	8.75 ± 1.24	1087 ± 218	124
**33**	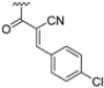	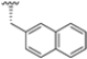	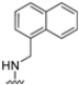	1.74 ± 0.15	387 ± 34	222
**34**	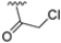	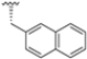	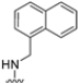	1.07 ± 0.23	572 ± 57	535
**35**	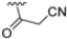	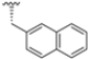	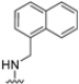	16.84 ± 2.24	869 ± 94	52

**Table 4:** Structure and IC_50_ values of active inhibitors **42**–**44** against β5i.

Cmpd.	Structure	β5i IC_50_ (μM)	β5c IC_50_ (μM)	β5i Selectivity (β5c/β5i Ratio)
**42**	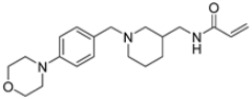	71 ± 10	38 ± 8	0.5
**43**	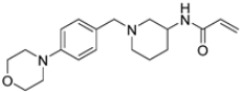	13 ± 2	25 ± 8	1.9
**44**	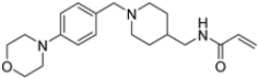	6 ± 1	4 ± 0	0.7

**Table 5:** IC_50_ values for β5i subunits of benzoxazoles-2(*3H*)-thione derivates **45**–**53**.

Cmpd	Structure	β5i IC_50_ (μM)	% Inhibition on β5c	β5i Selectivity (β5c/β5i Ratio)
**45**	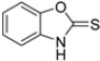	17	100%	n.d.
**46**	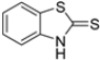	13	85%	n.d.
**47**	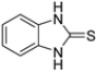	114	100%	n.d.
**48**	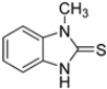	94	91%	n.d.
**49**	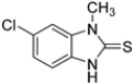	4.2 ± 0.9	94%	n.d.
**50**	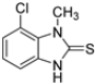	1.8 ± 0.3	99%	n.d.
**51**	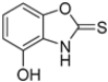	4.1 ± 0.9	87%	n.d.
**52**	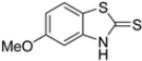	7.2 ± 6.1	n.d.	n.d.
**53**	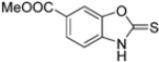	3.0 ± 1.3	n.d.	n.d.

n.d. = not determined due to a single-dose measurement of the activity on cCP.

**Table 6:** Structures and IC_50_ values of 1,3-benzothiazole derivates **54**–**57**.

Cmpd	Structure	β5i IC_50_ (μM)	β5c IC_50_ (μM)	β5i Selectivity (β5c/β5i Ratio)
**54**	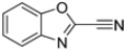	83 ± 6.0	82%	n.d.
**55**	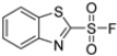	11 ± 2.0	81 ± 4.1	7.4
**56**	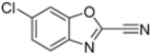	9.1 ± 4.5	45 ± 17	5
**57**	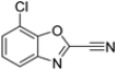	10 ± 4.6	93 ± 34	9.3

n.d. = not determined due to a single-dose measurement of the activity on cCP.

**Table 7:** Structures and IC_50_ values of novel benzoxazole-2-carbonitriles **58**-**61**.

Cmpd	Structure	β5i IC_50_ (μM)	β5c IC_50_ (μM)	β5i Selectivity (β5c/β5i Ratio)
**58**	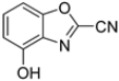	3.6 ± 2.8	30 ± 12	8.3
**59**	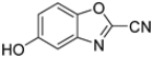	4.2 ± 2.5	53%	n.d.
**60**	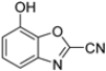	2.1 ± 1.2	11 ± 9	5.2
**61**	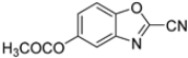	7.4 ± 1.7	55%	n.d.

n.d. = not determined due to a single-dose measurement of the activity on cCP.

**Table 8:** Biological activity of bortezomib, ixazomib, carfilzomib and compound **64** against the β5 subunits of the immunoproteasome and constitutive proteasome.

Cmpd	β5i IC_50_ (nM)	β5c IC_50_ (nM)	β5i Selectivity (β5c/β5i Ratio)
Bortezomib	1.3 ± 0.9	3.2 ± 1.5	2.5
Ixazomib	2.9 ± 2.3	5.0 ± 2.5	1.7
Carfilzomib	3.2 ± 1.1	2.1 ± 0.9	0.6
**64**	1.7 ± 0.7	41 ± 16	24

**Table 9:** Biological activity and selectivity of derivatives **65**–**69**.

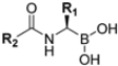					
Cmpd	R_2_	R_1_	β5i IC_50_ (nM)	β5c IC_50_ (nM)	β5i Selectivity (β5c/β5i Ratio)
**65**	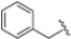	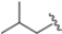	1000 ± 120	15,000 ± 3450	15
**66**	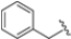	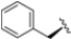	160 ± 340	16,000 ± 1910	99
**67**	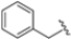	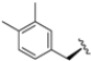	85 ± 2.8	15,000 ± 707	182
**68**	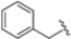	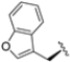	2.1 ± 1.0	900 ± 420	419
**69**	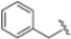	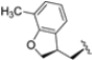	3.1 ± 0.7	7200 ± 495	2334

**Table 10:** Biological activity against β5i and β5c subunits of compound **70–78**.

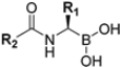					
Cmpd	R_2_	R_1_	β5i IC_50_ (nM)	β5c IC_50_ (nM)	β5i Selectivity (β5c/β5i Ratio)
**70**	** 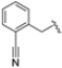 **	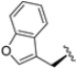	5.2 ± 0.6	1700 ± 71	334
**71**	** 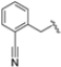 **	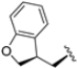	3.1 ± 0.8	2400 ± 669	758
**72**	** 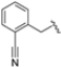 **	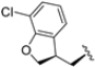	2.9 ± 0.3	7400 ± 1270	2573
**73**	** 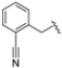 **	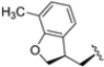	4.4 ± 0.5	14,000 ±1410	3147
**74**	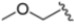	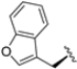	6 ± 2.0	820 ± 35	137
**75**	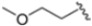	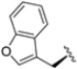	1.9 ± 0.4	400 ± 71	205
**76**	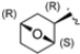	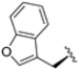	3.6 ± 2.4	2500 ± 396	684
**77**	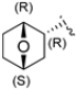	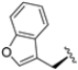	89 ± 17.9	15,000 ± 3310	173
**78**	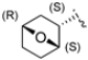	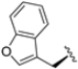	140 ± 21	>30,000	>210
